# Antioxidant Potential of Cookies Formulated with Date Seed Powder

**DOI:** 10.3390/foods11030448

**Published:** 2022-02-03

**Authors:** Zein Najjar, Jaleel Kizhakkayil, Hira Shakoor, Carine Platat, Constantinos Stathopoulos, Meththa Ranasinghe

**Affiliations:** 1 Department of Food Science, College of Food and Agriculture, United Arab Emirates University, Al-Ain P.O. Box 15551, United Arab Emirates; zeinrnajjar@gmail.com (Z.N.); 201990013@uaeu.ac.ae (M.R.); 2 Department of Nutrition and Health, College of Medicine and Health Sciences, United Arab Emirates University, Al-Ain P.O. Box 15551, United Arab Emirates; JaleelK@uaeu.ac.ae (J.K.); 201890012@uaeu.ac.ae (H.S.); PlatatCarine@uaeu.ac.ae (C.P.); 3 Faculty of Agrobiology, Food and Natural Resources, Czech University of Life Sciences Prague, 165 00 Prague, Czech Republic

**Keywords:** antioxidant, cookies, date seed, flavonoids, polyphenols

## Abstract

Utilising major waste products from the food industry can have both a great environmental impact and be a means to improve consumer health. Date seed is a food industry byproduct that has been proven to have high nutritional value. The aim of this work was to measure the total polyphenolic content (TPC), flavonoids, and antioxidant activity of the seeds of six date fruit varieties, *Fard*, *Khalas*, *Khinaizi*, *Sukkary*, *Shaham*, and *Zahidi*, and to use those seeds to enhance the antioxidant value of cookies by partially substituting flour with ground date seed. Date seed powder (DSP) was extracted at three levels of sample to solvent ratio (5:1, 10:1 and 15:1 mg/mL). Cookies were prepared using three substitution levels of wheat flour (2.5, 5.0, and 7.5%, *w*/*w*) by DSP and two types of flour (white and whole wheat), and were baked at two different temperatures, 180 and 200 °C. The composite cookies were found to contain a significant amount of TPC and flavonoids, and showed increased antioxidant activity compared with the control samples.

## 1. Introduction

The date palm tree is widely grown in regions of southwest Asia and North Africa [1,2,3], with more than 600 varieties currently cultivated [3,4]. The edible part of date fruit contains high amounts of sugars and dietary fibres as well as small amounts of protein, fat, ash, and polyphenols [5]. The inedible part, the date seed, represents a major waste in the date processing industry accounting for more than 6% of the fruit [6]; it has significant amounts of fibres, minerals, vitamins, lipids and proteins [6,7], and is rich in antioxidants [1,6,7]. Date seed has been proven to have the potential to ameliorate liver damage and to protect against hepatotoxicity in rats [8].

Baked foods are popular among consumers because of their taste and widespread availability in the form of biscuits, cookies, muffins, cakes and more. Cookies and biscuits are among the most consumed bakery products, as they are ready to eat, cheap, and available in a wide range of flavours [9,10,11,12,13]; on the other hand, as with most bakery items, they are high in sugar and have low amounts of antioxidants, fibre, and minerals [14]. Consumers today tend to choose and eat healthier food [15,16], and there is an increasing trend to make cookies and biscuits utilising functional ingredients [12,13,17]. The use of refined flour results in products lacking the nutritive value of grain in terms of dietary fibres and phytochemicals [18,19]. At the same time, achieving the sensory parameters of cookies (taste, texture and colour) that meet consumers expectations [20], especially while avoiding the use of synthetic additives, can be challenging [21]. Antimicrobials, antioxidants, and anti-browning agents are currently used in the food industry as preservatives [22]. It has been reported that the use of synthetic molecules may be linked to carcinogenesis, and this has led to some restraints on their use [23,24,25]; synthetic preservatives can be replaced by natural extracts from plant origin, which provide bioactive properties and thus increase the nutritive value of the final product [26,27,28]. Natural extracts from spices, fruit powder, and aromatic plants for antioxidant purposes have been incorporated in bakery, dairy, and meat products [20,25,29,30,31]. As the food industry generates high amounts of by-products rich in valuable constituents which can be utilised as functional ingredients, such as antioxidants, incorporating these substances in food would be beneficial environmentally and economically while providing healthier options to consumers [32]. Consumer demand towards functional foods enriched with by-products [33] is increasing due to enhanced awareness of the health and environmental implications of current food choices. Hence, development of cookies fortified with date seeds, the major by-product of the date industry, can generate significant impact environmentally through the reduction of waste financially through the development of a new product and the reduction of waste disposal costs, and societally through the enhanced nutrition made available to consumers.

The objective of this study, therefore, is to quantify the total phenolic and flavonoid content and the antioxidant activity of six varieties of date seed powder (DSP), and to evaluate these characteristics in composite cookies formulated with partial substitution of wheat flour by DSP. 

## 2. Materials and Methods

### 2.1. Chemicals

Follin–Ciocalteu’s reagent, gallic acid, 2,2-diphenyl-1-picrylhydrazyl (DPPH), 6-hydroxy-2,5,7,8-tetramethylchroman-2-carboxylic acid (Trolox), 2,2′-azinobis-3-ethylbenzothiazoline-6-sulfonic acid (ABTS), glacial acetic acid, hydrochloric acid, ferric chloride, 2,4,6-tripirydyl-S-triazine, aluminium chloride, sodium hydroxide, quercetin (Sigma-Aldrich, Steinheim, Germany), sodium carbonate, sodium phosphate, methanol, ethanol, potassium persulfate, sodium acetate trihydrate, and sodium nitrite (Merck, Darmstadt, Germany) were used; all chemicals were of analytical grade.

### 2.2. Date Seed Powder Production

The seeds of six varieties of Emirati dates, *Fard*, *Khalas*, *Khinaizi*, *Sukkari*, *Shaham*, and *Zahidi* (2 kg of each), were obtained from local farms in Al Ain, United Arab Emirates. Date seeds were soaked in water for six hours, air-dried for three days, ground using a Teeba Date Seed Grinding Machine (Teeba Engineering Industries LLC, Dubai, United Arab Emirates) and sieved to obtain date seed powder with particles of less than 250 µm diameter.

### 2.3. Production of Formulated Cookies with 2.5%, 5%, and 7.5% Date Seed Powder

Cookie dough was made according to the AACC method [34] with some modifications. Ingredients were acquired from the local market (80 g white/whole wheat flour, 30 g palm oil (raised to 40 g when using whole wheat flour), 35 g sucrose, 0.8 g NaHCO_3_, 1.0 g salt, 0.4 g NH_4_HCO_3_, and 17.6 g water). Cookies were produced by replacing 2.5%, 5%, and 7.5% (*w*/*w*) of flour with date seed powder. Two types of flour (white, WF, and whole wheat, WW) were used to make cookie dough, which was kneaded to a uniform 3 mm thickness with the aid of a pasta machine and cut with a stainless-steel circular cutter of 4.5 cm diameter. The cookies were baked at two different temperatures (A: 180 °C for 10 min; B: 200 °C for 8 min), making twelve combinations for each date seed variety. Baked cookies were packed in sealed plastic bags and stored at −20 °C until extraction.

### 2.4. Sample Preparation and Extraction

#### 2.4.1. Powder

Powder was extracted at three sample to solvent ratios, 5:1, 10:1, and 25:1, following the procedure of Khanavi et al. [35]. Specifically, 25, 50, and 75 mg of date seed powder were mixed with 5 mL (40–50 °C) of deionised water. The mixture was placed on a shaker for 5 min and left at 4 °C for 30 min prior to 10 min centrifugation at 10,000× *g*, the supernatant was collected, and one additional extraction was made with 4 mL (40–50 °C) deionised water. The supernatants were pooled and stored at −20 °C until analysis.

#### 2.4.2. Cookie

Composite cookies (1 g) were mixed with 5 mL of solvent (deionised water) [35]. Composite cookies were prepared at three levels of substitution of flour by DSP (2.5%, 5%, 7.5%). The mixture was placed on a shaker for 5 min and left at 4 °C for 30 min prior to 10 min centrifugation at 10,000× *g*, the supernatant was collected, and one additional extraction was made with 4 mL (40–50 °C) deionised water. The supernatants were pooled and stored at −20 °C until analysis.

### 2.5. Determination of TPC and Flavonoids 

TPC was determined using Folin–Ciocalteu reagent and external calibration with gallic acid as mentioned in [36] with some modifications; 20 µL of the sample was diluted in 180 µL deionised water and mixed with 200 µL Folin–Ciocalteu reagent, vortexed, and 800 µL sodium carbonate was added and incubated for 30 min at 40 °C. Absorbance was measured at 765 nm. The concentrations of TPC were calculated from the standard curve of gallic acid in the range of 0 to 25 µg/mL; the results are expressed as Gallic acid equivalent in (µg/g) of the sample.

Flavonoids were measured by the aluminium chloride colorimetric method as described in [37]; in a vial, 150 µL of the sample was mixed with 150 µL methanol and 75 µL 5% NaNO_2_ and allowed to react for 5 min at room temperature. Then, 1.25 mL 7% AlCl_3_ and 0.5 mL 5% NaOH were added and left for an extra 5 min, and absorbance was measured at 510 nm. The concentrations of flavonoids were calculated from the standard curve of quercetin in the range of 25 to 200 µg/mL, with the results expressed as a percentage (*w*/*w*). 

### 2.6. Determination of Antioxidant Activity

The DPPH scavenging ability of the extracts was determined according to the modified method of [38]; 100 µL of sample was mixed with 400 µL 0.1 mM DPPH, and free radical scavenging activity was determined by measuring the absorbance at 520 nm after 30 min of incubation at room temperature.

ABTS scavenging ability was determined according to the procedure developed in a previous study [39]. Specifically, 5 mL of 7 mM ABTS was added to 88 μL of 140 mM potassium persulphate solution and left to react for 24 h at room temperature in the dark. Then, ABTS solution was diluted (1:88) with 10 mM sodium phosphate buffer of pH 7.4. A 600 μL volume of the ABTS was added to the 50 μL of the sample. Absorbance was measured at 734 nm after 5 s of incubation at room temperature. The results were expressed as mmol of Trolox equivalents per mg of sample.

A modified assay method of the Ferric-reducing antioxidant power (FRAP) reported in [40] was performed; 100 µL of the sample was mixed with a prewarmed mixture of 500 µL 300 mM acetate buffer (pH 3.6), 50 µL 10 mM 2,4,6-tripirydyl-S-triazine, and 50 µL 20 mM FeCl_3_, incubated at 37 °C for 5 min, and absorbance was measured at 593 nm.

### 2.7. Statistical Analysis

All experiments were performed with more than three replications; the results obtained are expressed as mean ± standard deviation (SD). Data were assessed by one way ANOVA followed by Tukey’s test using Minitab 19, State College, Pennsylvania, USA statistical software package; *p* < 0.05 determined the level of significance.

## 3. Results and Discussion

### 3.1. Analysis of Date Seed Powder

**Figure 1:**
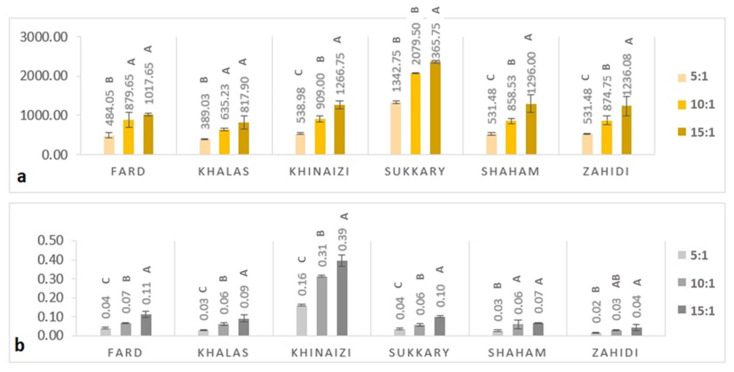
(**a**) TPC (Gallic acid equivalent per gram (µg/g) of the sample); (**b**) Flavonoid content (*w*/*w*) % of six varieties of date seed powder extracted at three sample to solvent ratios (5:1, 10:1, and 15:1). Different uppercase superscript letters in a variety denote significant differences, *p* < 0.05.

The results regarding TPC are presented in Figure 1a. TPC measurements ranged from 389.03 to 2365.75 µg GAE/g at 5:1 and 15:1 sample to solvent ratio levels, respectively. The data clearly show that higher sample to solvent ratios had a higher content of polyphenols. The results of our study did not correlate with previous investigations of the TPC of certain date seed varieties. The TPC of *Khalas* seed was 2194 µg/g [41]; for *Khinaizi* and *Zahidi* it was 9540 and 11,610 µg/g [42], respectively. For *Sukkary* it was 37.1 µg/g [43], while in another study it was 2058 µg/g for *Zahidi* [44]. This variation appears related to different extraction methods [42], and the use of water as opposed to other solvents in extraction, which has been proven to have low solubility for phenolics and flavonoids [1]. For example, in Ardekani et al., higher TPCs were detected when the polar aprotic solvent dimethyl sulfoxide (DMSO) was used in comparison to common polar solvents (water, aqueous methanol, and methanol) [42]. This underlines the importance of the extraction solvent when determining TPC.

Figure 1b presents the data on flavonoid content, which ranged from 0.02 to 0.39 (*w*/*w*) %, and the *Khinaizi* variety had not less than three-fold higher the amount of flavonoid content among the other varieties, with values of 0.16, 0.31, and 0.39 (*w*/*w*) % at 5:1, 10:1, and 15:1 sample to solvent ratio levels, respectively. At the same time, *Zahidi* had the lowest flavonoid content with 0.04 (*w*/*w*) %, at the highest sample to solvent ratio level. Though the TPC was high in *Sukkary*, flavonoid content was comparatively lower than *Khinaizi*.

The variations in TPC and flavonoid content in date seed powder might be due to differences in date seed variety, maturity, growing conditions, season, fertiliser, soil type and storage conditions [42,45]. For instance, higher contents of phenolics were reported in the seeds of Omani date fruits [45], whereas much lower amounts were shown in Saudi Arabian varieties [43]. Ahfaiter et al. showed variances in flavonoid content in *Sukkary* date seed powder ranging from 1.2–1.4%, depending on the geographical origin [46]. Apart from this, the reason for the observed differences in results from previous studies is the difference in the extraction procedures used [42,47]. Several previous studies used several solvents for extraction, especially organic solvents or organic and aqueous solution mixtures. For example, Parry et al. used acetone for pumpkin seed flour [48], whereas Mistrello et al. [44] used an acetone and water mixture for date seeds. Several studies have used aqueous methanol or ethanol for extraction of seed flour from date seeds, mango seeds, and sesame seeds [46,49,50,51,52]. In our study, even with water as the solvent the extraction efficiency was significant, showing considerable amounts of phenolic compounds in alignment with Al Juhaimi et al. [43] and Ifesan et al. [11], who used aqueous extracts of seed flour as well.

**Figure 2:**
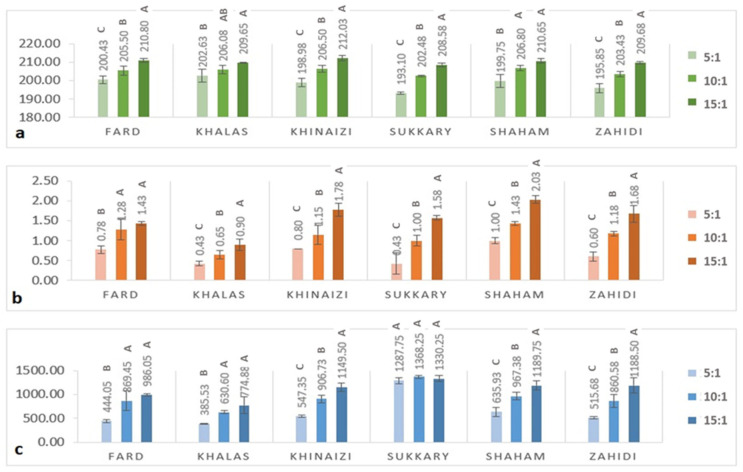
(**a**) DPPH radical scavenging activity (µg Trolox equivalent/g sample); (**b**) ABTS radical scavenging (% Scavenging effect with ABTS/mg) %; (**c**) Ferric reducing assay, (µg equivalent Trolox/g) of six varieties of date seed powder extracted at three sample to solvent ratios (5:1, 10:1, and 15:1). Different uppercase superscript letters in a variety denote significant differences, *p* < 0.05.

The antioxidant properties in terms of radical scavenging activity of date seed powder using DPPH and ABTS assay and antioxidant power using FRAP assay are shown in Figure 2. In DPPH assay (Figure 2a), the data range from 193.1 to 212.03 µg/g of date seed powder, and in ABT from 0.43 to 2.03 % per mg of date seed powder. At the same time, in FRAP the range was from 385.53 and 1368.25 µg/g. The phenol and flavonoid assays showed similar trends, the with higher sample to solvent ratio levels being correlated with higher antioxidant activity. It was noted that the *Sukkary* variety, shown in Figure 1a and Figure 2c, had the highest phenolic content and the highest FRAP values among all varieties by a significant margin. However, although the *Sukkary* variety had the highest FRAP activity, a plateau was observed in that above a 5:1 sample to solvent ratio level there was no increase.

The majority of the total phenolic content was assumed to be composed of flavonoids, with 0.39% at the highest sample to solvent ratio level. These dietary phenolics may be the ones most responsible for the high antioxidant capacity reported in date seeds with increasing sample to solvent ratio levels. Hence, this suggests that date seeds can be considered a potential raw material as a natural, active ingredient for food applications such as bakery products. As explained in Al-Farsi et al. [45], when compared to the other by-products of dates, the seeds have the highest antioxidant activity thanks to their high phenolic content.

### 3.2. Total Phenolic Content (TPC) and Flavonoid Content of Composite Cookies

**Figure 3:**
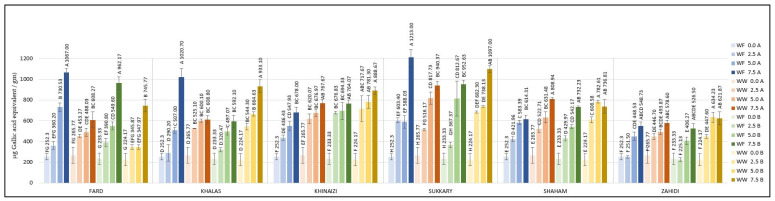
TPC of composite cookies formulated with 2.5%, 5%, and 7.5% substitution of flour by date seed powder. (Data expressed as Gallic acid equivalent per gram (µg/g) of the cookie sample. Different uppercase superscript letters in a variety denote significant differences, *p* < 0.05. (A—180 °C, B—200 °C).

The TPC of cookies enriched with date seed powder was measured; the contents are listed in Figure 3. The highest TPC of the control cookies (zero date seed powder substitution) was 265.77 µg/g. Almost all the composite cookie formulations showed higher TPC levels above 265.77 µg/g, except *Zahidi* with 2.5% substitution level of white flour at both 180 and 200 °C temperature conditions. Cookies formulated with 7.5% *Fard* variety made with white flour at either temperature had significantly higher TPC content, more than 962.17 µg/g, and at substitution level of 7.5% the TPC level were more than three-fold higher compared to the control cookies. The TPC of *Khalas* cookies at 7.5% substitution level was the highest when made with white flour and baked at 180 °C and with whole wheat flour baked at 200 °C. The values of *Khinaizi* and *Shaham* cookies were mostly similar. The highest value of TPCs, observed in *Sukkary* date seed powder (Figure 1a) at 7.5% substitution level, explains the highest values of *Sukkary*-made cookies, which range from 940.37 to 1213.00 µg/g, almost four times higher than control cookies, and which differs from the TPC of *Zahidi*-made cookies, which presented the lowest effect among all varieties with value of 621.87 µg/g when made with 7.5% substitution level, whole wheat flour and baked at 200 °C. Some previous studies on the total phenolic content of date seed powder exhibited high polyphenol content, for example, in *Sukkary* [43,44,46], strengthening the results of this study.

**Figure 4:**
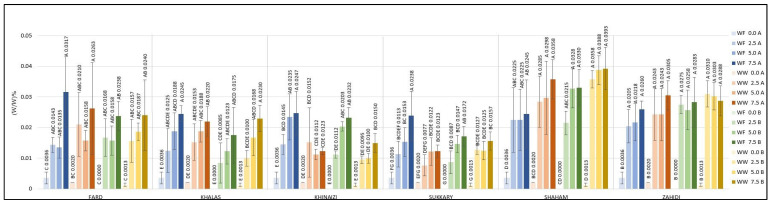
Total contents of Flavonoids of composite cookies formulated with 2.5%, 5%, and 7.5% substitution of flour by date seed powder. (Data expressed as Gallic acid equivalent per gram (µg/g) of the cookie sample. Different uppercase superscript letters in a variety denote significant differences, *p* < 0.05. (A—180 °C, B—200 °C).

On the other hand, it was expected that the high flavonoid content found in *Khinaizi* date seed powder (Figure 1b) would lead to *Khinaizi* cookies having the highest values (Figure 4); instead, composite cookies of *Shaham* and *Zahidi* had the highest content of flavonoids, up to 0.0393 (*w*/*w*) %. Cookies formulated with *Fard*, *Khalas*, *Khinaizi*, and *Sukkary* had mostly similar measurements, ranging from 0.0077 (*w*/*w*) % at 2.5% substitution level to 0.0317 (*w*/*w*) % at 7.5%. A similar pattern was observed between *Sukkary* composite cookies and *Sukkary* flour observations; i.e., despite having the highest TPC level, the flavonoid content was comparatively lower than the other date seed varieties, although higher than the control cookies. Though the TPC and flavonoid content of cookies increased with higher levels of date seed flour, a substantial decrease in those levels in cookies in comparison to flour was observed upon baking. The reason behind this could be attributed to the loss of phenolic components during baking [53].

However, it is clearly shown that the increase in TPCs and flavonoids was noticeable regardless of the flour type and baking temperature. Both polyphenols and flavonoids have a strong contribution to human health. Polyphenols are known to have antioxidant activity, anti-inflammatory actions and anticarcinogenic activities [54,55] while flavonoids have the ability to interfere with the formation and propagation of free radicals and protect low density lipoproteins from oxidation [56]. The variability of their therapeutic potential depends on the structure of the particular flavonoid [57,58,59]. The results indicate that date seeds can be effectively used to enhance the antioxidant potential of cookies.

### 3.3. Antioxidant Activity

**Figure 5:**
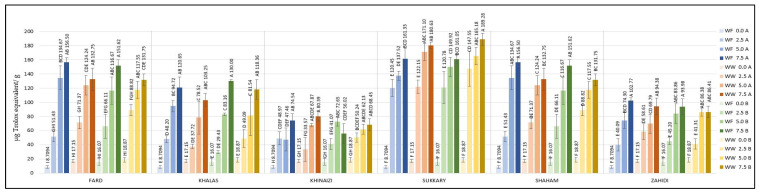
DPPH radical scavenging activity (µg Trolox equivalent/g) of composite cookies formulated with 2.5%, 5%, and 7.5 % substitution of flour by date seed powder Different uppercase superscript letters in a variety denote significant differences, *p* < 0.05. (A—180 °C, B—200 °C).

**Figure 6:**
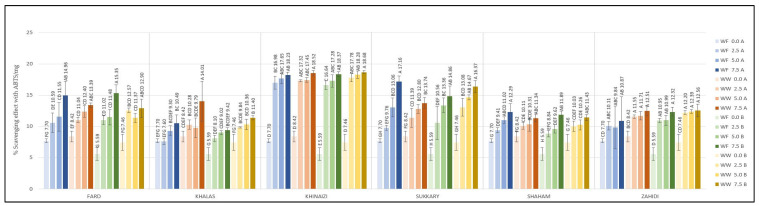
ABTS radical scavenging (% Scavenging effect with ABTS/mg) of composite cookies formulated with 2.5%, 5%, and 7.5 % substitution of flour by date seed powder. Different superscript letters in a variety denote significant differences, *p* < 0.05. (A—180 °C, B—200 °C).

Cookies produced at substitution levels of 2.5%, 5% and 7.5% were tested for their radical scavenging activity using the DPPH and ABTS assays. The DPPH results are shown in Figure 5; the data show that substitution of either type of flour resulted in increased antioxidant activity in the cookies. Similar results can be seen in the data from the ABTS assay, shown in Figure 6.

Regarding DPPH, the control cookies had values of less than 18.87 µg/g, and even the cookies with the lowest substitution level had values more than four-fold higher than the control, except for *Khalas*- and *Khinaizi*- made cookies (regardless of flour type and temperature), where the values only double. Even though *Sukkary* date seed powder was insignificantly different from other varieties the *Sukkary* cookies had the highest antioxidant activity, with more than 161.05 µg/g at 7.5% substitution level, while *Khinaizi* cookies had the lowest at all substitution levels, with no more than 80.39 µg/g, which is opposite from the ABTS results. *Khinaizi* cookies had the highest measurements, with more than 18.23% ABTS/g at the highest substitution level, slightly higher than *Sukkary* cookies (17.16% ABTS/g). Mistrello et al., Al Juhaimi et al., and Ardekani et al., showed high antioxidant activity in *Sukkary* date seed powder, where it seems that it contributes to the high antioxidant activity in composite cookies as well [43,44,46]. Very slight differences were observed between the *Fard*, *Khalas*, *Shaham* and *Zahidi* varieties; ABTS ranged from 7.6% to 12.7%, 9.02% to 12.40%, and from 9.42% to 15.35% ABTS/g at 2.5%, 5.0%, and 7.5% respectively.

**Figure 7:**
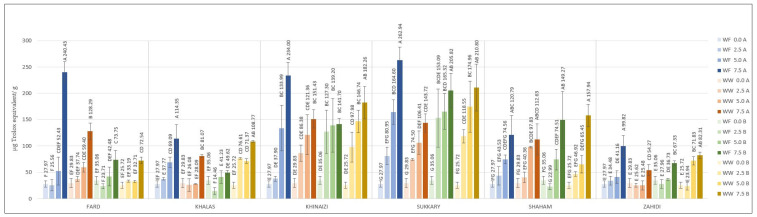
Ferric reducing antioxidant power, (µg equivalent Trolox/g) of composite cookies formulated with 2.5%, 5%, and 7.5 % substitution of flour by date seed powder. Different superscript letters in a variety denote significant differences, *p* < 0.05 (A—180 °C, B—200 °C).

The antioxidant power of the cookies was determined using the FRAP procedure (Figure 7). The data show that the maximum antioxidant power was achieved at 7.5% substitution level of date seed powder, with the highest value of 262.94 µg/g for *Sukkary* composite cookies. *Fard* substitution was affected by baking temperature; cookies baked at 180 °C have 240.43 and 128.29 µg/g using white flour and whole wheat flour, respectively, compared with less than 73.75 µg/g for cookies baked at 200 °C, while the temperature did not influence FRAP results of the other varieties. On the other hand, the *Khinaizi* and *Sukkary* varieties exhibited higher FRAP values regardless of the flour type and baking temperature, and similar values to *Fard* cookies at 7.5% substitution and 180 °C baking temperature. Almost all of the varieties showed the highest reducing antioxidant power with white flour at 7.5% substitution level and baking temperature at 180 °C, except for the *Shaham* composite cookies. Although FRAP value increased with increasing levels of date seed powder in the cookies, the values were comparatively lower than the FRAP values of flour. The antioxidant capacity is based on preventing free radical formation by breaking free radical chain reactions through donation of hydrogen atoms [60]. As the polyphenols are responsible for this reaction, the loss of polyphenols might be the reason for this observation of reduced FRAP values upon baking, as there was a greater reduction in antioxidant power at the higher temperature of 200 °C. Overall, while an increase in antioxidant capacity was observed upon substitution of date seed flour, the increment was lower at the higher temperature.

The differences in antioxidant levels obtained from the assays might be a reflection of a relative difference in the activity of antioxidant compounds in the extract [61]. Date seeds contain very high levels of phenolic antioxidants [42,43,44,45]. Because date seeds are rich in dietary fibre [45,62,63,64,65], they are considered a good source of natural antioxidants due to their richness in phenolic compounds. Mrabet et al.’s explanation of date dietary fibre concentrate indicates the presence of significant amount of bound phenolics in dietary fibre [66], which adds additional health benefits to the antioxidant properties of date seed. In this study, water was used as the extraction solvent for extraction of TPCs and for assays of antioxidant action, ABTS, and FRAP, yet the results were significantly higher. Most of the studies on bakery items incorporated seeds including grape seed, flaxseed, sunflower seed and fig seed, and used organic solvents such as ethanol and methanol [32,67,68,69,70,71], ending up with high levels of TPCs, antioxidants, ABTS and FRAP levels.

High levels of antioxidants in date seed powder as well as the improved antioxidant properties of composite cookies with increasing substitution levels show that date seed powder can be used as functional ingredient in the food industry to improve the quality of baked goods. Our results show that the substitution of flour with date seed powder significantly upgraded the content of polyphenols and flavonoids as well as the antioxidant activity. A number of studies have reported similar results regarding the increased nutritive value of composite bakery products, including cookies and biscuits fortified with several seed varieties. For example, Acun and Gul [13] observed high antioxidant and TPC in cookies formulated with grape seed flour, while Kaur et al. found rich antioxidant capacity which was evident from higher TPCs [67]. Another study showed enhanced antioxidant potential and flavonoid levels in cookies formulated with chia and quinoa seed flour [12]. Aksoylu et al. [68] found higher TPC in biscuits fortified with blueberry and grape seeds, while Gbenga-Fabusiwa et al. [72] and Grasso et al. [14] reported higher phenolic and antioxidant levels in biscuits incorporated with pigeon-pea and sunflower seed flour, respectively, than the biscuits made only with wheat flour.

As wheat flour has a very low polyphenol content [73], incorporation of date seed powder would be expected to increase the nutritional value of bakery products such as cookies. Moreover, increasing the natural antioxidant content in bakery products leads to the extension of shelf life by lowering the fat oxidation, which is a factor determining food quality [74]. The obtained results reveal the importance of incorporating date seed flour to achieve better functionality of produced cookies.

## 4. Conclusions

This study investigated date seed powder to determine its natural polyphenolic compound and flavonoid contents and showed it to have high antioxidant potential. The highest amount, 2365.75 µg GAE/g TPC at a 15:1 sample to solvent ratio level, was in *Sukkary* date seed powder, while *Khinaizi* exhibited the highest flavonoid content. Mostly similar DPPH values were observed in all the varieties, with the highest value at 15:1 sample to solvent ration level. *Shaham* seed flour showed a higher ABTS activity, whereas *Sukkary* was significantly higher in FRAP, having quite similar values in all three sample to solvent ration levels. Cookies formulated with 7.5% *Fard* variety made with white flour at either temperature had a significantly higher TPC content, more than 962.17 µg/g. Although the highest flavonoid content was found in *Khinaizi* date seed powder, composite cookies of *Shaham* and *Zahidi* had high flavonoid contents of up to 0.0393 µg/g as well. The increase in TPCs and flavonoids was observable regardless of the flour type and baking temperature. Both DPPH and FRAP results showed the highest antioxidant activity in *Sukkary* cookies at 7.5% substitution level, with amounts of 189.28 µg/g at 200 °C and 262.94 µg/g at 180 °C, respectively. *Khinaizi* cookies had the highest ABTS measurements, with more than 18.23% ABTS/g at 7.5% substitution level, slightly higher than *Sukkary* cookies with 17.16% ABTS/g. Moreover, higher TPCs and flavonoids resulted even under less efficient conditions of extraction where water was used as the solvent.

Based on the results for the composite cookies in this study, it can be concluded that the TPC and antioxidant activity of date seeds successfully enhanced the quality of final product; hence it can be recommended as a flour fortifier and as an ingredient in functional foods. These findings improve our knowledge on the value of utilising date seeds, which are considered a waste product in the food industry. Future work could focus on clinical trials where conclusive observations can be made as to how these fortified cookies might positively impact consumer health.

## Data Availability

The datasets generated for this study are available on request to the corresponding author.
